# EEG-Driven Prediction Model of Oxcarbazepine Treatment Outcomes in Patients With Newly-Diagnosed Focal Epilepsy

**DOI:** 10.3389/fmed.2021.781937

**Published:** 2022-01-03

**Authors:** Bin Wang, Xiong Han, Zongya Zhao, Na Wang, Pan Zhao, Mingmin Li, Yue Zhang, Ting Zhao, Yanan Chen, Zhe Ren, Yang Hong

**Affiliations:** ^1^Department of Neurology, Zhengzhou University People's Hospital, Zhengzhou, China; ^2^Department of Neurology, Henan Provincial People's Hospital, Zhengzhou, China; ^3^School of Medical Engineering, Xinxiang Medical University, Xinxiang, China; ^4^Department of Neurology, Henan University People's Hospital, Zhengzhou, China

**Keywords:** precision medicine, machine learning, prediction model, gradient boosting decision tree (GBDT) model, EEG complexity

## Abstract

**Objective:** Antiseizure medicine (ASM) is the first choice for patients with epilepsy. The choice of ASM is determined by the type of epilepsy or epileptic syndrome, which may not be suitable for certain patients. This initial choice of a particular drug affects the long-term prognosis of patients, so it is critical to select the appropriate ASMs based on the individual characteristics of a patient at the early stage of the disease. The purpose of this study is to develop a personalized prediction model to predict the probability of achieving seizure control in patients with focal epilepsy, which will help in providing a more precise initial medication to patients.

**Methods:** Based on response to oxcarbazepine (OXC), enrolled patients were divided into two groups: seizure-free (52 patients), not seizure-free (NSF) (22 patients). We created models to predict patients' response to OXC monotherapy by combining Electroencephalogram (EEG) complexities and 15 clinical features. The prediction models were gradient boosting decision tree-Kolmogorov complexity (GBDT-KC) and gradient boosting decision tree-Lempel-Ziv complexity (GBDT-LZC). We also constructed two additional prediction models, support vector machine-Kolmogorov complexity (SVM-KC) and SVM-LZC, and these two models were compared with the GBDT models. The performance of the models was evaluated by calculating the accuracy, precision, recall, F1-score, sensitivity, specificity, and area under the curve (AUC) of these models.

**Results:** The mean accuracy, precision, recall, F1-score, sensitivity, specificity, AUC of GBDT-LZC model after five-fold cross-validation were 81%, 84%, 91%, 87%, 91%, 64%, 81%, respectively. The average accuracy, precision, recall, F1-score, sensitivity, specificity, AUC of GBDT-KC model with five-fold cross-validation were 82%, 84%, 92%, 88%, 83%, 92%, 83%, respectively. We used the rank of absolute weights to separately calculate the features that have the most significant impact on the classification of the two models.

**Conclusion:** (1) The GBDT-KC model has the potential to be used in the clinic to predict seizure-free with OXC monotherapy. (2). Electroencephalogram complexity, especially Kolmogorov complexity (KC) may be a potential biomarker in predicting the treatment efficacy of OXC in newly diagnosed patients with focal epilepsy.

## Introduction

Epilepsy is a chronic disease affecting more than 70 million people worldwide, it is characterized by recurrent, paroxysmal, rigid, and unpredictable alterations of sensory and motor systems, and abnormal electrical activity of neurons (1, 2). Epilepsy is classified into four types: focal, generalized, combined generalized and focal, and unknown onset (3). Focal epilepsy, accounting for 60% of all epilepsies, is the most frequent type of epilepsy and occurs in patients of all ages (4). Comparative monotherapy trials in patients with newly diagnosed focal epilepsy have shown that oxcarbazepine (OXC) is equal in efficacy to phenytoin and immediate-release carbamazepine but may have superior tolerability (5–7). Pharmacotherapy is the primary treatment modality for epilepsy, however, in some patients, seizures cannot be controlled with antiseizure medicine (ASM) and lead to significant risks of neuronal damage and cognitive decline (8). This highlights a need for the prediction of drug response at the drug initiation phase.

However, ASM response is complex and is modulated by multiple factors, including environmental, anthropometric, and genetic factors, and biological subsystems affected by the disease (9). The current standard of care relies on trial and error with sequential therapy. Although there are drug selection guidelines based on seizure types (focal or generalized onset), many drugs have similar efficacy (10). So, drug selection becomes extremely difficult as it is impossible to predict which drugs will be the most effective in a particular patient. There are also no biomarkers that can reliably predict treatment response during conventional treatment.

Since the 1980s, precision medicine has emerged as a new paradigm for improving and promoting patient-specific medicine. Its key goal is to provide personalized treatment for every patient where medical decisions are based on the individual characteristics of the patient, rather than the average characteristics of the entire patient population. Precision medicine requires the analysis of different types of multivariate data from the same individual. It has been used in the early diagnosis and prevention of diseases, reduction of the risk of side effects and adverse events of medications, and in the design of clinical trials (11, 12). The development of precision medicine is inseparable from artificial intelligence. Machine learning, as a branch of artificial intelligence, has the ability to build integrated and multi-scale models by integrating different types of features at different levels. Recently, De Jong et al. (13) integrated pharmacogenetics and clinical data to achieve an accurate prediction of brivaracetam treatment response, but this method is not cost-effective to be integrated in the clinical practice (14).

Studies have shown that the Electroencephalogram (EEG) signal is an internal “fingerprint” of individuals (15). Although with the increase of age, EEG frequency, amplitude, and other aspects will change to a certain extent, the oscillation network of brain waves in each adult brain is relatively stable, and many genetic, structural, and functional abnormalities related to diseases are more or less directly involved in the generation and/or synchronization of brain wave oscillations. Electroencephalogram can be used as a biomarker for the treatment of brain diseases such as epilepsy (16). Other studies and our previous work have shown that it is possible to predict ASM response using EEG-based artificial intelligence (17–20). So, here we test whether the integration of EEG and clinical data can be used to construct a prediction model of OXC treatment outcomes, that can facilitate the correct selection of ASM in newly-diagnosed patients with focal epilepsy patients.

## Materials and Methods

### Participants and Data Acquisition Participants

The retrospective study was approved by the Henan Provincial People's Hospital ethics committees, and informed consent was obtained from all participants. Six thousand three hundred seventy patients with epilepsy were registered between January 2014 and April 2021 at the Epilepsy Center of Henan Provincial People's Hospital. Focal epilepsy is defined as seizures originating within networks limited to one hemisphere and the seizures may be discretely localized or widely distributed (21). Patients who meet the following criteria were included: newly diagnosed focal epilepsy with drug-naïve; OXC is the only ASM after diagnosis; long-term scalp EEG recordings were conducted before drug initiation; more than 1 year of follow-up. Exclusion criteria were the following: generalized epilepsy and epileptic syndrome; other ASM were taken before OXC; the combination of other ASMs; lack of EEG data; follow-up data of <1 year and poor adherence; pregnant or lactating women.

**Figure 1:**
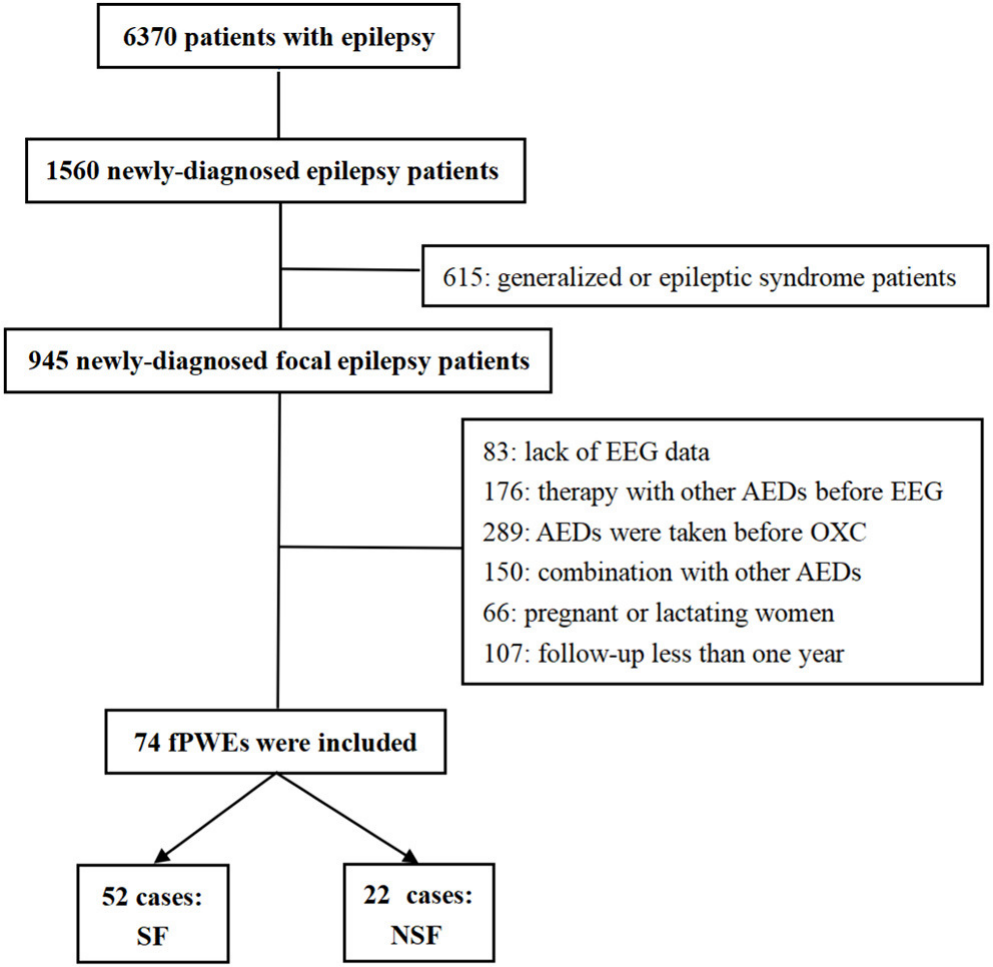
Flow chart. EEG, electroencephalogram; AEDs, antiepileptic drugs; OXC, oxcarbazepine; fPWEs, patients with focal epilepsy; SF, seizure-free; NSF, not seizure-free.

Seventy-four individuals with newly diagnosed patients with focal epilepsy patients, initially treated with OXC, were enrolled at our center. After 1 year of follow-up, according to Engel class (22), SF was defined as patients with epilepsy who met Class I while not seizure-free (NSF) was defined as patients who met Engel Class II, III, and IV. Finally, 52 patients were enrolled in the SF group and 22 in the NSF group (Figure 1).

### Clinical Data

We included 15 clinical features: sex, age, age at the onset of the disease, follow-up time, seizure frequency before OXC, seizure circadian rhythm, comorbidities, inducement, history of perinatal injury, physical development, family history of epilepsy, MRI, temporal lobe epilepsy (TLE), history of central nervous system infection, and history of head injuries (23–26) (Table 1).

**Table 1:** Demographical and clinical status of the participants.

	**SF (*n* = 52)**	**NSF (*n* = 22)**	***x*** **^2^** **/*Z*/*t*** **-value**	***P-*** **value**
Sex (Male/female)	33/19	11/11	1.162^a^	0.281
Age, year	14.5 ± 11.50	16.50 ± 12.00	−0.681^b^	0.496
Age at onset, year	13.5 ± 12.25	15.50 ± 13.38	−0.361^b^	0.718
Follow-up time, months	32.58 ± 9.83	36.18 ± 8.91	−1.481^c^	0.143
Seizure frequency before OXC, times/month	0.65 ± 0.70	15.50 ± 13.37	−1.983^b^	0.045^*^
Seizure circadian rhythm (day/night/both)	19/16/17	8/7/7	0.009^a^	0.995
Comorbidity (Y/N)	27/25	16/6	2.749^a^	0.097
Inducement (Y/N)	29/23	17/5	3.039^a^	0.081
History of perinatal injury (Y/N)	12/40	12/10	6.986^a^	0.008^*^
Physical development (N/AN)	9/43	3/19	4.469^a^	0.035^*^
Family history (Y/N)	2/50	2/20	0.832^a^	0.362
MRI(P/N)	17/35	13/9	4.469^a^	**0.035** ^*^
TLE(Y/N)	16/36	8/14	0.221^a^	0.638
History of CNS infection (Y/N)	7/45	2/20	0.261^a^	0.599
History of head injury (Y/N)	5/47	2/20	0.005^a^	0.944

*SF, seizure-free; NSF, not seizure-free; OXC, oxcarbazepine; Y/N, yes/no; N/AN, normal/abnormal; P/N, positive/negative; MRI, magnetic resonance imaging; TLE, temporal lobe epilepsy; CNS, central nervous system*.

a *For qualitative data, Chi-square tests were used*.

b *For quantitative data, after Shapiro-Wilk normality test, the Mann-Whitney U-test was applied for data with abnormal distributions, data that did not conform to normal distributions were presented as the median ± interquartile range*.

c *Data with a normal distribution were compared by the independent sample t-tests, mean ± standard deviation was used to describe. p < 0.05 is considered as statistically significant*.

* *Defined as features that have statistically significant between SF group and NSF group*.

*Bold values are statistically significant*.

### EEG Data

#### EEG Acquisition

Long-term scalp EEG was carried out by EEG-1200C electroencephalograph (Nihon Kohden, Tokyo, Japan), the sampling rate was 256 Hz, the amplifier was 1,000x. Electrodes were placed according to the international 10–20 system. There were 19 scalp electrodes and 2 reference electrodes. The discharges of EEG were marked independently by two experienced electroencephalographers. If there were any disputes, another clinical neurologist was consulted. We intercepted a continuous 1-h EEG including the waking period and the sleeping period. The waking period and the sleeping period accounted for 30 min each.

#### EEG Preprocessing

Matlab software (Mathworks Inc., USA) equipped with the EEGLAB toolbox was used for EEG preprocessing (27). Electroencephalogram preprocess was as follows: firstly, 0.5–30 Hz EEG fragments were retained using bandpass filter. Then independent component analysis was used to remove artifacts. Next, EEG data without epileptic charges or artifacts, were taken while the patient was awake, and their eyes were open. The EEG data were divided into 15 s periods and recalculated based on a reference average. Finally, 15 time periods for each subject were randomly selected for subsequent analysis.

#### EEG Complexity Estimators

##### Lempel–Ziv Complexity

Lempel–Ziv complexity (LZC) is a simple non-parametric measure to calculate the randomness of a one-dimensional finite-length sequence. It was related to the number of different substrings and their occurrence rate along the sequence. The larger the value is, the more complex the corresponding data is (28, 29). The following procedure was done with MathWorks in MATLAB. Before calculating EEG complexity, the binarization was performed according to the median value of the EEG time series. For binary sequences S (S1, S2,..., S*n*), the sequence length is *n*, and *c*(*n*) is defined as the LZC value of the EEG time series. When a new subsequence appears in the time series, *c*(*n*) increases by one unit, and the pattern search continues until the last string is scanned. For a sufficiently long random 0–1 sequence, the following formula holds if 0 and 1 are equally likely to occur:


(1) $$\begin{array}{r} \underset{n\to \infty }{\lim}c\left(n\right)\;=\;b\left(n\right)\;=\;n/\log2^{n} \end{array}$$

The *b*(*n*) is used to normalize *c*(*n*) to obtain a value independent of the sequence length *n*, so LZC is:


(2) $$\begin{array}{r} LZC\;=\;c\left(n\right)/b\left(n\right) \end{array}$$

##### Kolmogorov Complexity

Kolmogorov complexity (KC), known as algorithmic complexity, is defined as a new algorithmic measure of randomness for generating quantitative definitions of information (30). Kolmogorov complexity describes the randomness of an object, which is a string based on the length of a computer program; the complexity of a string, consisting of 0 and 1, is estimated by the number of bits of the shortest computer program that produces the string. The KC is described as follows:


(3) $$\begin{array}{r} k_{u}\left(x\right)\;=\;\min p:u\left(p\right)\;=\;x^{l\left(p\right)} \end{array}$$

Where *p* is the computer program and *l*(*p*) is the length of *x* output strings of *u* general Turing machine (computer). Kolmogorov complexity is the minimum length of the output of a computer program. To calculate the KC of an EEG, the data were first converted into discrete binary sequences. Then, KC estimation methods could be used to analyze the bits of the shortest computer program associated with the discrete sequence. Based on previous reports, the KC estimation was carried out by the difference method. When the difference between two sequential samples was positive, the method assigned 1, and when the difference was negative, it assigned 0.

### Model Process

For imbalance in a sample, SMOTE was used to strike an equilibrium during the training process (31). Toolkits: Python's sklearn toolkits (32). To avoid overfitting, default parameters were used unless otherwise specified. The parameters were set as follows: The number of nearest neighbors is 5 (*K* = 5); Degree of over-sampling: making the number of positive and negative samples consistent. SMOTE in this paper was done independently within each training set, not used before cross-validation. The SMOTE consists of two functions, SMOTE (*T, N, K*) and Populate (*N, i, nnarray*). The SMOTE code idea is very simple: scan every sample point, calculate *K* nearest neighbor of every sample point, record the index of each nearest neighbor point in *nnarray*, then pass it into Populate (*N, i, nnarray*), and complete a sample point. Populate is responsible for randomly generating *N* samples similar to the observed sample *i* based on the index in the *nnarray*. The function calculates the gap dif between random neighboring point *nn* and each feature of observed sample point *i*, multiplying the gap by a [0,1] random factor gap, and then combining the value of dif ^*^ gap plus the observation point *i* (33, 34).

#### SVM Model

The support vector machine (SVM) is a classical classifier with good performance in dichotomies (35). It has good performance for small samples (36). Lib-SVM was used for the classification process. The core of SVM is to establish an optimized hyperplane. A linear SVM classifier was constructed based on kernel parameter and regularization C parameter. In this study, the C parameter was set to 1.

The five-fold cross-validation was used for the classification process, four-fold pats as the training sets, and one-fold part as the validation sets. This process was repeated five times until all subjects went through it once. The recursive feature elimination (RFE) was used for feature selection. In cross-validation, the RFE occurs in the training sets but not the validation sets, with the results of the RFE feature screening from the training sets to guide the feature selection in the validation sets. Absolute weight value was applied to the feature selection procedure; the greater the absolute weight of features, the greater the influence on classification. We used EEG complexity features such as KC and LZC combined with clinical features to establish two SVM-RFE models for predicting SF with OXC monotherapy in patients with newly diagnosed focal epilepsy.

#### GBDT Model

Based on the theory of Gradient boosting machine (GBM), Gradient Boosting Decision Tree (GBDT) is a typical representative of ensemble learning, which is a lifting algorithm (37). Gradient Boosting Decision Tree can effectively avoid overfitting by combining decision trees with gradient algorithms. It is considered that all machine learning algorithms can be used as the basic learning machine of gradient lifting by GBM. Because decision trees are easier to understand and calculate compared with other algorithms, GBDT chooses decision tree as the base learning machine. Decision tree can combine multiple features, and has good processing ability for non-parameterized features. Therefore, when there are outliers or non-linearly separable data in the data, decision tree can be used to process these data. However, the decision tree suffers from the drawback of overfitting. So, combining the decision tree (formed by the combination of multiple gradient lifting methods) with the gradient lifting algorithm can reduce the overfitting of the decision tree (38, 39).

Two predictive models were established by GBDT using KC-clinical, LZC-clinical characteristics. Five-fold cross-validation was also used for this process. In this process, absolute weights were used to rank the features that influence classification, as well.

### Descriptive Statistics

Statistical analysis was calculated with SPSS. The Shapiro-Wilk normality test was used to assess the normality distribution of data. For quantitative data, the independent sample *t*-tests were used to compare the data with a normal distribution (Mean ± standard deviation); the Mann-Whitney U-test was applied for data with abnormal distributions (median ± interquartile range). A strict false discovery rate based on the Benjamini–Hochberg correction was applied to *p*-values to correct for multiple comparisons. While, for qualitative data, Chi-square tests were used. *P* < 0.05 was considered statistically significant in this study.

## Results

The study included 44 males and 30 females. The age range was 5–70, the overall follow-up time ranged from 12 to 60 months. The average follow-up time was 21.97 months in 74 individuals-−24.17 months in the SF group and 25.13 in the NSF group. There were no significant differences in gender, age, and follow-up time between the two groups. However, significant differences were found in seizure frequency before OXC (*p* = 0.045), history of perinatal injury (*p* = 0.008), physical development (*p*=0.035), and MRI (*p* = 0.035) (Table 1).

### GBDT-LZC

**Figure 2:**
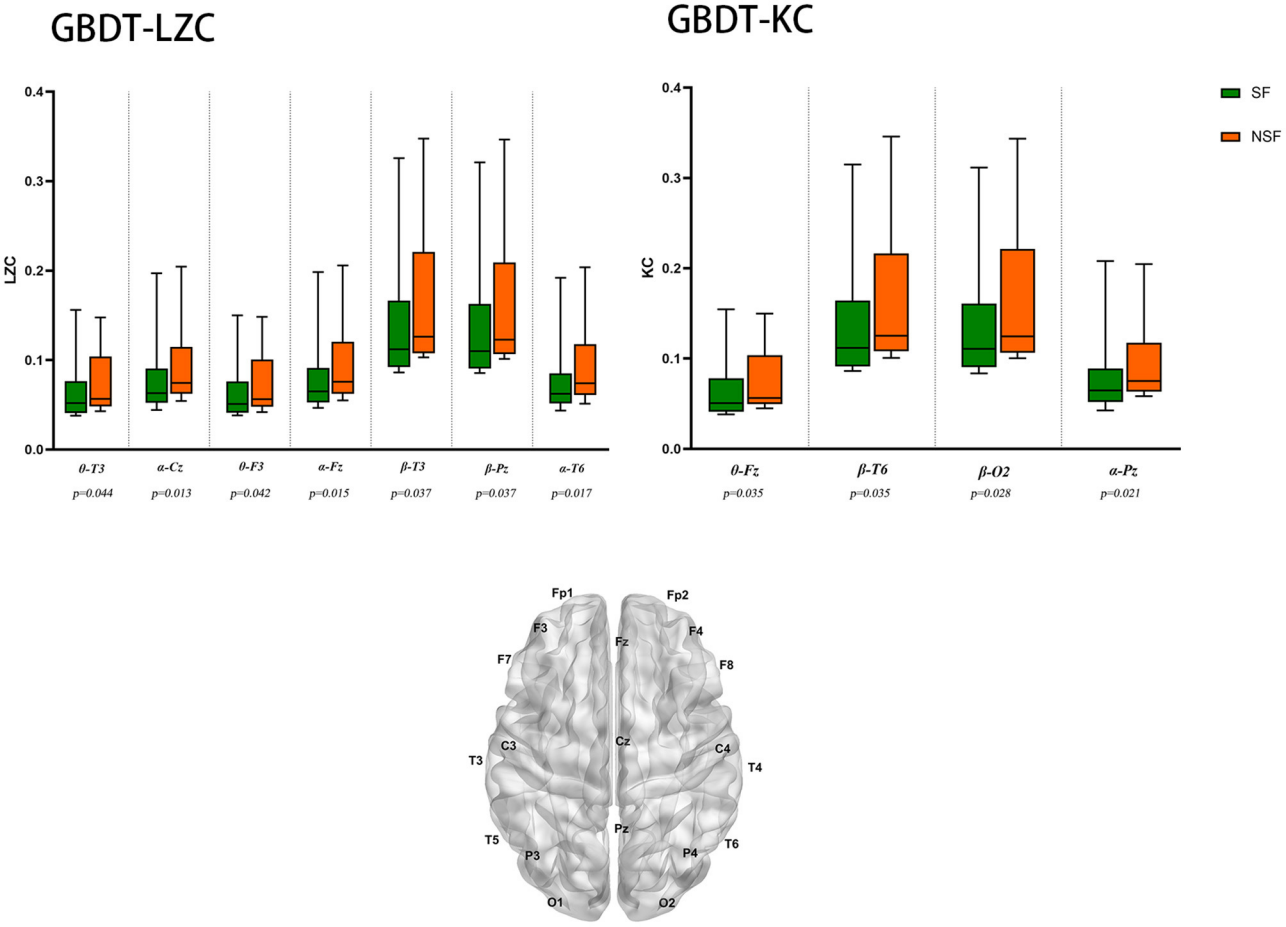
EEG features that have the most significant impact on classification. θ-T3: θ band from T3 channel, *p* < 0.05 is considered statistically significant.

**Figure 3:**
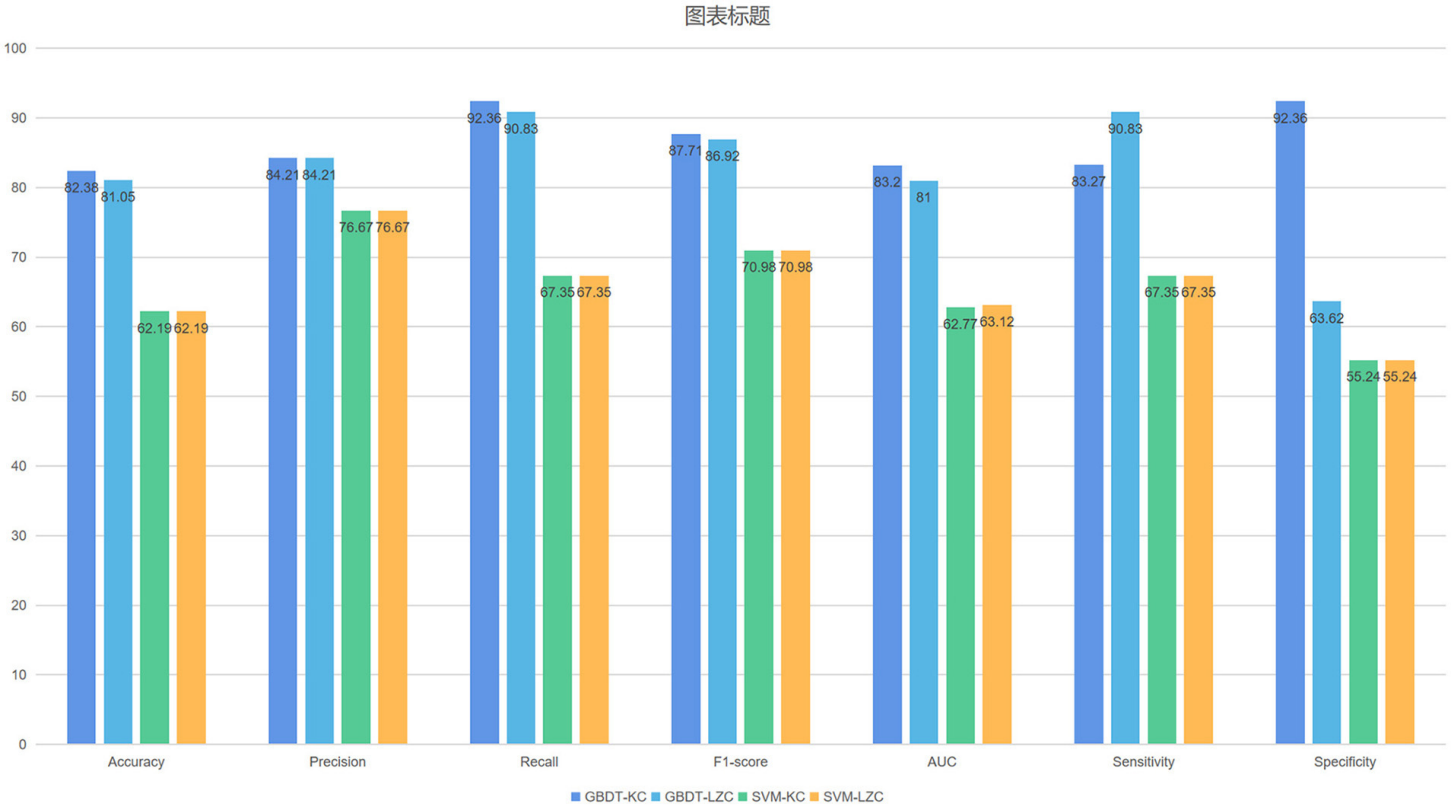
The mean evaluation indexes after five-fold cross-validation.

The NSF group showed higher LZC than the SF group. The top 10 features that influenced classification were δ band from F8 channel, θ band from T3 channel (*p* < 0.05), α band form Cz channel (*p* < 0.05), θ band from F3 channel (*p* < 0.05), α band form Fz channel (*p* < 0.05), θ band from T6 channel, TLE, β band from T3 and Pz channel (*p* < 0.05), α band form T6 channel (*p* < 0.05) (Table 2A; Figure 2). The mean accuracy, precision, recall, F1-score, sensitivity, specificity, and AUC of the GBDT model after five-fold cross-validation were 81%, 84%, 91%, 87%, 91%, 64%, 81%, respectively (Table 3A; Figure 3). The mean accuracy, precision, recall, F1-score, sensitivity, specificity, and AUC of the SVM-RFE model after five-fold cross-validation were 62%, 77%, 91%, 87%, 91%, 64%, 81%, respectively (Table 3B; Figure 3).

**Table 2:** The top 10 features that impacting the GBDT classifier mostly.

	**SF**	**NSF**	***Z/t*** **value**	***P*** **-value**	***P*** ^′^ **-value**
**(A) GBDT-LZC**					
δ-F8	0.0240 ± 0.0214	0.0298 ± 0.0132	−1.656	0.098	0.098
θ-T3	0.0518 ± 0.0356	0.0566 ± 0.0558	−2.010	0.044	0.060
α-Cz	0.0631 ± 0.0383	0.0745 ± 0.0500	−2.472	0.013	**0.032** ^*^
θ-F3	0.0510 ± 0.0352	0.0561 ± 0.0526	−2.032	0.042	0.057
α-Fz	0.0649 ± 0.0384	0.0757 ± 0.0582	−2.424	0.015	**0.036** ^*^
θ-T6	0.0531 ± 0.0394	0.0587 ± 0.0528	−1.880	0.060	0.068
TLE					
β-T3	0.1119 ± 0.0742	0.1261 ± 0.1133	−2.081	0.037	0.050
β-Pz	0.1100 ± 0.7230	0.1227 ± 0.1024	−2.081	0.037	0.050
α-T6	0.0624 ± 0.0334	0.0742 ± 0.0569	−2.389	0.017	**0.038** ^*^
**(B) GBDT-KC**					
δ-T3	0.2245 ± 0.0068	0.2531 ± 0.2333	−1.809	0.070	0.070
θ-F7	0.0503 ± 0.0204	0.0575 ± 0.0555	−1.904	0.057	0.065
Seizure frequency before OXC					
θ-FP1	0.0521 ± 0.038	0.0573 ± 0.0566	−1.928	0.054	0.061
θ-T6	0.0537 ± 0.0398	0.0594 ± 0.0535	−1.904	0.057	0.065
θ-Fz	0.0505 ± 0.0371	0.0564 ± 0.0540	−2.105	0.035	**0.044** ^*^
β-T6	0.1116 ± 0.0728	0.1250 ± 0.1084	−2.105	0.035	**0.044** ^*^
β-O2	0.1109 ± 0.0704	0.1244 ± 0.1148	−2.200	0.028	**0.031** ^*^
Seizure circadian rhythm					
α-Pz	0.0645 ± 0.037	0.0753 ± 0.0542	−2.306	0.021	**0.028** ^*^

*GBDT, gradient boosting decision tree; LZC, Lempel-Ziv complexity; KC, Kolmogorov complexity; SF, seizure-free; NSF, not seizure-free; TLE, temporal lobe epilepsy; δ-F8, δ band from F8 channel; OXC, oxcarbazepine; P′-value refers to P-value that is corrected by false discovery rate correction*.

* *The features that have statistically significance. Although the selected features may not be statistically significant, they did have a classification value in the model*.

*Bold values are statistically significant*.

**Table 3:** The performance of the four classifier models.

	**Fold1**	**Fold2**	**Fold3**	**Fold4**	**Fold5**	**Mean-Value**
**(A) GBDT-LZC**						
Accuracy (%)	67	87	80	93	79	81
Precision (%)	69	80	81	100	90	84
Recall (%)	90	100	90	92	82	91
F1-score (%)	78	89	86	96	86	87
AUC (%)	64	89	82	100	70	81
Sensitivity (%)	90	100	90	92	82	91
Specificity (%)	20	71	60	100	67	64
**(B) SVM-LZC**						
Accuracy (%)	60	53	67	67	64	62
Precision (%)	70	56	78	100	80	77
Recall (%)	70	63	70	62	73	67
F1-score (%)	70	59	74	76	76	71
AUC (%)	54	64	64	100	33	63
Sensitivity (%)	70	63	70	62	73	67
Specificity (%)	40	43	60	100	33	55
**(C) GBDT-KC**						
Accuracy (%)	67	87	80	100	79	82
Precision (%)	69	80	82	100	90	84
Recall (%)	90	100	90	100	82	92
F1-score (%)	78	89	86	100	86	88
AUC (%)	66	89	88	100	73	83
Sensitivity (%)	66	89	88	100	73	83
Specificity (%)	90	100	90	100	82	92
**(D) SVM-KC**						
Accuracy (%)	60	53	67	67	64	62
Precision (%)	70	56	78	100	80	77
Recall (%)	70	63	70	62	73	67
F1-score (%)	70	59	74	76	76	71
AUC (%)	54	63	64	100	33	63
Sensitivity (%)	70	63	70	62	73	67
Specificity (%)	40	43	60	100	33	55

*LZC, Lempel-Ziv complexity; GBDT, gradient boosting decision tree; AUC, the area under the curve; SVM, support vector machine; RFE, recursive feature elimination; KC, Kolmogorov complexity*.

### GBDT-KC

**Figure 4:**
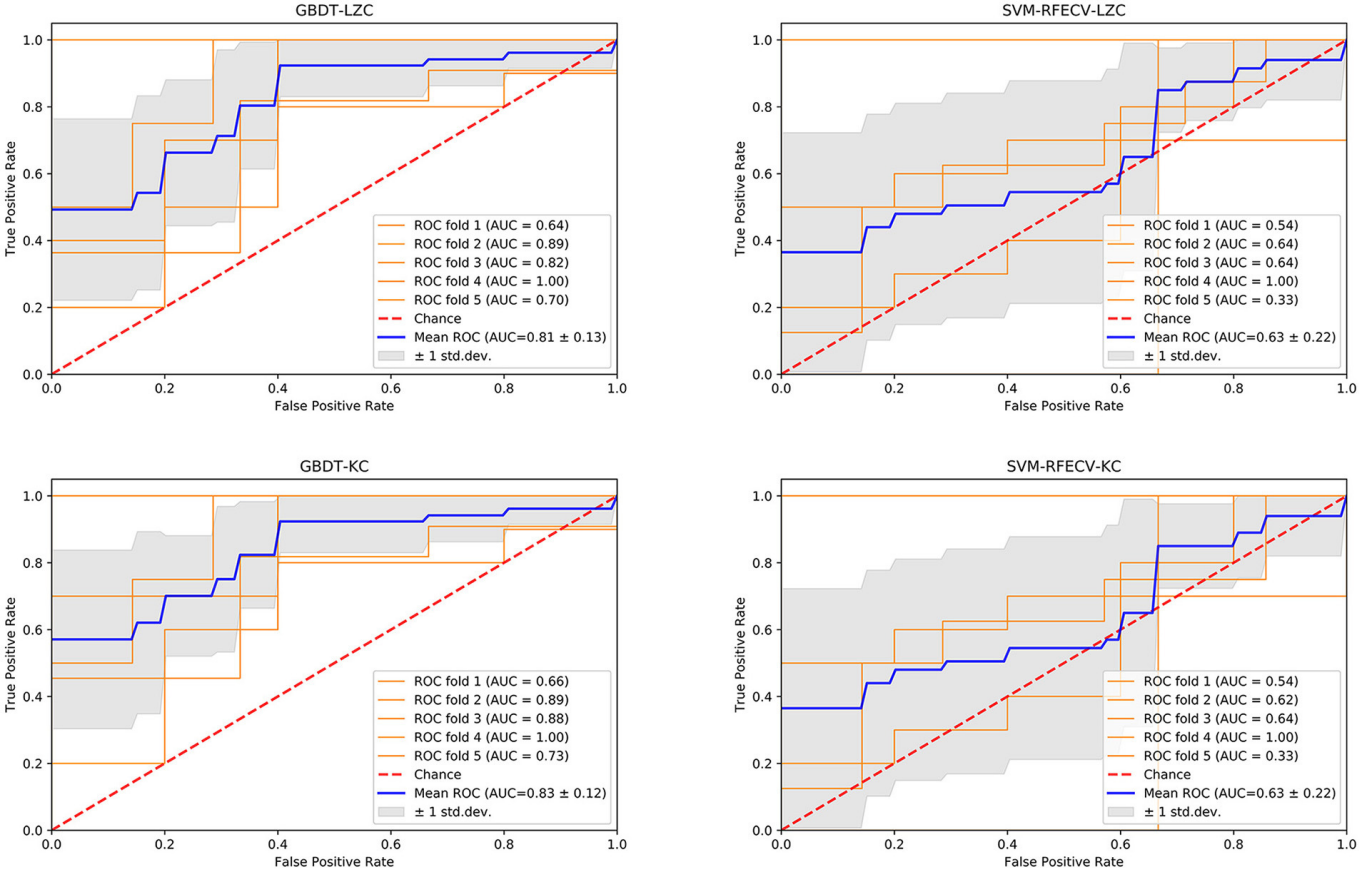
The performance of four models. GBDT, gradient boosting decision tree; LZC, Lempel-Ziv complexity; AUC, area under the curve; ROC, receiver operating-characteristic curve; std. dev, standard deviation; SVM, support vector machine, RFE, recursive feature elimination, CV, cross-validation; KC, Kolmogorov complexity.

Like LZC, it was apparent that the NSF group has higher KC than the SF group. The top ten features that have the highest absolute weights were δ band from T3 channel, θ band from F7 channel, seizure frequency before OXC, θ band from FP1 channel, T6 and Fz (*p* < 0.05) channel, β band from T6 and O2 channel (*p* < 0.05), seizure circadian rhythm, and α band form Pz channel (*p* < 0.05) (Table 2B; Figure 2). Although the selected features may not be statistically significant, they did have a classification value in the model. The model yielded average accuracy of 82%, precision of 84%, recall of 92%, F1-score of 88%, sensitivity of 83%, specificity of 92%, and AUC of 83% after five-fold cross-validation, respectively (Table 3C; Figure 3). Compared with the GBDT model, SVM-RFE model yielded mean accuracy, precision, recall, F1-score, sensitivity, specificity, AUC of five-fold cross-validation were 62%, 77%, 67%, 71%, 67%, 55%, 63%, respectively (Table 3D; Figure 3). The results of each fold were presented in Figure 4.

## Discussion

We constructed a model for predicting OXC treatment outcomes. Our GBDT-KC model (EEG complexity and clinical data) performed better in terms of the performance merits compared with De Jong's study (pharmacogenetics and clinical data) (13). Our research has a more clinical application because it is cost-efficient. To our knowledge, this is the first study that applied EEG complexity to predict OXC response in patients with focal epilepsy, and achieved good performance.

### EEG Complexity as a Biomarker for Epilepsy

Electroencephalogram plays an important role in the diagnosis, treatment, and prognosis of epilepsy (40–42). Electroencephalogram signals have non-linear structures in the time dimension. Recently, new methods for studying EEG signals have been developed from non-linear systems theory since non-linear measurements are more suitable to reflect the complex, irregular, and non-stationary behavior of neural processes. The non-linear analysis quantifies the complexity of EEG and reflects the state of brain neural networks. Electroencephalogram complexity correlates with synchronization (43); highly synchronized signals (e.g., epileptic seizures) give rise to low complexity values (44). Complexity is related to the degree of entropy, so some of these estimators are called entropy estimators. Based on the complexity of the algorithm, LZC does not rely on large amounts of EEG data and is suitable for short and non-stationary time series (45). Kolmogorov complexity is defined as the complexity of a sequence and is based on the length of the shortest program that could generate the sequence (46). Kolmogorov complexity was found to be more sensitive to the detection in patients with schizophrenia compared with other measures (47). However, the application of EEG complexity in epilepsy remains limited.

In our study, the LZC and KC showed a complexity decrease in the SF group compared with the NSF group. Though the relationship between EEG complexity and epilepsy is not clear, EEG complexity is related to the severity and prognosis of the disease. Cerquera et al. (48) analyzed the difference between cognitive deficit schizophrenia (DS) and non-cognitive deficit schizophrenia (NDS), and found that the DS group showed less LZC in the frontal lobe than the NSD group. Another study found a significant reduction in EEG complexity, 2 min before the seizure, compared with the inter-seizure period (about 6–8 min before the seizure) (49). Valproic acid treatment also decreased the overall complexity of 19 EEG channels in patients with idiopathic epilepsy (50).

### The Prediction Models of Drug Response

Antiseizure medicines are still the mainstream treatment for patients with epilepsy. Non-standard treatment in the early stage has been shown to contribute to poor prognosis (51). Therefore, it is necessary to choose appropriate ASMs for epileptic patients. Although there are many ASMs for focal epilepsy, the differences between these ASMs are unclear and many ASMs are cross-referenced for both focal and generalized epilepsy. Further, there are different adverse effects and different treatment responses of these ASMs. Therefore, ASMs suitable for patient A, may not be the right choice for other patients (B, C,…). Previous studies of drug response were based mostly on clinical characteristics, without an in-depth analysis of individual patients (52, 53).

Recently, precision medicine has made significant development where the goal is to make personalized medical decisions based on the individual characteristics of the patient. Precision medicine is closely related to pharmacogenetics, but mostly in oncology, and has considerable impact on drug prescription (54). Outside oncology, genetic information has not yet played a major role in drug selection. However, the field is an active area of research. Precision medicine is usually associated with gene manipulation or gene targeting. De Jong et al. (13) designed a phase III clinical trial in which 235 participants were randomly assigned to brivaracetam or placebo groups. Only the genomes of brivaracetam treated patients were sequenced. Clinical characteristics and whole genome sequencing (WGS) pharmacogenetics data were combined to predict brivaracetam drug response in patients with focal epilepsy. The GBDT classifier was confirmed as the best performing model with an AUC of 0.76 in the discovery datasets and 0.75 in the validation datasets; the asymptotic 95% confidence interval (CI) was wide (0.6–0.9). However, the study had several limitations which are as follows: the dimensions of WGS data are huge, concerns over overfitting, WGS data were generated only for patients with brivaracetam therapy, the 95% CI in validation datasets as wide and it was too expensive to be clinically applicable. Our GBDT model, based on clinical and EEG complexity features, achieved better prediction performance than De Jong's study, with an average AUC of 0.832. At present, using EEG to predict drug response has high clinical value, the price is more affordable, and the prediction performance is not bad. Although, pharmacogenetics is not cost-effective currently, if the cost of genetic sequencing decreases and/or the demonstrated benefit of genetically-guided ASM selection is increased, then it may be cost-effective. It cannot be denied that EEG combined with pharmacogenetics would be more clinically beneficial.

Lin et al. (18) used 24 univariate EEG features extracted from EEG fragments from 11 drug-refractory epilepsy patients and 16 control epilepsy patients to predict drug-resistant epilepsy; the study yielded a precision rate of 0.942, ROC area 0.938. We have devised an integrated model combining clinical features and EEG functional connectivity. Our study used the phase lag index functional connectivity to predict drug-refractory epilepsy for newly diagnosed epilepsy patients and achieved good performance with AUC of 0.98, an accuracy of 0.94, sensitivity of 0.95, and specificity of 0.93(17). We show that EEG has good prediction performance in drug response.

Zhang et al. (20) used clinical and EEG sample entropy features to predict drug response with levetiracetam therapy via SVM achieved good performance. Our SVM-RFE model was inferior to Zhang's, while, the GBDT model achieved better performance than theirs'. In this study, we established a GBDT-KC model to predict SF for patients with focal epilepsy with OXC monotherapy. Our study yielded an average accuracy of 82%, a precision of 84%, recall of 92%, F1-score of 88%, sensitivity of 83%, specificity of 92%, and AUC of 83% after five-fold cross-validation, respectively. Focal-onset epilepsy accounts for the majority of all epilepsy cases. The selection of ASMs for focal epilepsy is of great clinical significance. However, there is still no referenced study for personalized drug selection for focal epilepsy. Our study may facilitate future studies in this field.

### Limitations and Prospects

There are limitations to our study. The study was retrospective, and selection bias is inevitable. Prospective studies need to be conducted in the future. The sample size in our study was small, and the model was only suitable for Asians. Multi-center studies with large sample sizes and diverse populations are required. Currently, the clinical problem is the choice between multiple potential ASMs. For example, there are many ASMs for focal epilepsy, such as carbamazepine, OXC, lamotrigine, valproate, clobazam, topiramate, phenytoin, phenobarbital, and zonisamide. Although this study was focused on the drug response of OXC, our final purpose was to make personalized and optimal treatment with less adverse effects for newly diagnosed epilepsy patients. We demonstrated that it is feasible to predict ASMs' response in combination with clinical and EEG complexity features, EEG complexity could be used as a biomarker to predict drug response, the concept is still in its theoretical stage. Our study proposed the possibility of this research in this area, there is still a long way to go in the future.

## Conclusion

We established a GBDT-KC prediction model for seizure outcome of patients with focal epilepsy with OXC monotherapy. EEG complexity, especially KC can be used as a biomarker for predicting outcomes of ASMs treatment.

## Data Availability Statement

The original contributions presented in the study are included in the article/supplementary material, further inquiries can be directed to the corresponding authors.

## Author Contributions

XH and NW obtained funding. BW designed the study, acquired the data, analyzed EEG recordings, worked on EEG preprocessing and machine learning process, drafted, and revised the manuscript. XH designed the study and revised the manuscript. ZZ aided assistant in machine learning process. PZ and ML analyzed EEG recordings. NW, TZ, and YC analyzed and interpreted the data. YZ, ZR, and YH conducted the statistical analysis. All authors revised this draft, read, and approved the final manuscript.

## Funding

This study was sponsored by Henan Province's Gong Jian Program (Authorization number: SB201901074), 23456 Talent Engineering (Authorization number: ZC20200371), and the National Natural Youth Fund (Authorization number: 81801291).

## Conflict of Interest

The authors declare that the research was conducted in the absence of any commercial or financial relationships that could be construed as a potential conflict of interest.

## Publisher's Note

All claims expressed in this article are solely those of the authors and do not necessarily represent those of their affiliated organizations, or those of the publisher, the editors and the reviewers. Any product that may be evaluated in this article, or claim that may be made by its manufacturer, is not guaranteed or endorsed by the publisher.

## Abbreviations

ASM: antiseizure medicine
CI: confidence interval
GBDT: gradient boosting decision tree
GBM: gradient boosting machine
KC: Kolmogorov complexity
LZC: Lempel-Ziv complexity
NSF: not seizure-free
OXC: oxcarbazepine
RFE: recursive feature elimination
SF: seizure-free
SVM: support vector machine
TLE: temporal lobe epilepsy
WGS: whole genome sequencing.
